# Risk factors of pilonidal sinus disease in preparatory school students; a case control study

**DOI:** 10.1016/j.amsu.2020.07.016

**Published:** 2020-07-15

**Authors:** Faruq H. Faraj, Hiwa O. Baba, Abdulwahid M. Salih, Fahmi H. kakamad

**Affiliations:** a College of Medicine,University of Sulaimani, Madam Mitterrand Street, Sulaimani, Kurdistan, Iraq; b Kurdistan Board for Medical Specialty, Iraq; c Smart Health Tower, Madam Mitterrand Street, Sulaimani, Kurdistan, Iraq; d Kscien Organization, Hamdi Street, Azadi Mall, Sulaimani, Kurdistan, Iraq

**Keywords:** Pilonidal sinus, PNS, Student, Risk factor

## Abstract

**Background:**

Pilonidal sinus disease (PNS) is a disorder of the young population. The aim of this study is to analyze the risk factors associated with development of PNS in the secondary school students.

**Methods:**

This case control study enrolled 189 participants, (Control group: 95 cases, case group: 94 cases). The inclusion criterion for the trial group was those secondary school students with PNS and age ranged between 16 and 20 years without the known risk factors of PNS.

**Result:**

About 80 (42.3%) patients were male and 109 (57.7%) were female. Each group included 95 patients. There was no significant difference in both groups regarding basic features. Among the control group 36 (37.9%) participants were used to study while sitting on a hard place whereas among the case group 62 (66%) cases were used to study while sitting on a hard place, the difference was statistically significant (<0.001).

**Conclusion:**

Sitting on the hard places could be regarded as a risk factor of developing PNS among secondary school students.

## Highlights

• Pilonidal sinus disease is a disorder of the young population.
• Several risk factors have been recognized like job, being hairy …. etc.
• Studying for prolonged time has never being studied to cause PNS.
• In this study, factors have been assessed to cause PNS in preparatory students.

## 1 Introduction

Pilonidal sinus disease is a disorder of the young population, which can lead to tract formation, pus collection and tenderness. Skin penetration by hair is the hallmark of the disease [1]. The foreign bodies and fragmented hairs accumulate in the moist regions of the body resulting in infection [2]. It restricts the daily activity of patients because of the pain, discharge and sometimes bad odder [3].

PNS is believed to be an acquired disorder rather than being a congenital one. It is frequently seen in the natal cleft and less frequently develops in other regions like umbilicus, interdigital webs, suprapubic area, nose, groin, axilla, penis, clitoris, occiput and prepuce [4,5].

About fifty percent of perianal disorders are PNS. Generally it develops in the age range of 10–40 years. Male gender is affected more than female by a ratio of 3:1. Risk factors include young age, long period of sitting, excessive hair, deep natal cleft and poor hygiene [4].

Regarding management, several noninvasive and invasive techniques like (lying open, incision and drainage, marsupialization, rhomboid excision or excision and primary closure, Limberg flap and modified Limberg flap) have been used. In spite of these procedures, the condition may result in recurrence and postoperative complications [6].

The aim of this study is to determine and discuss the risk factors associated with the development of PNS. The study work has been recorded in line with the STROCSS criteria [7].

## 2 Materials and method

**Registration and Ethics:** the study has been registered in accordance with the declaration of Helsinki (Every research study involving human subjects must be registered in a publicly accessible database before recruitment of the first subject). The study was registered in http://www.researchregistry.com, the registration number is researchregistry5642. It can be found on https://www.researchregistry.com/browse-the-registry#home/registrationdetails/5ecda096ab824500151b98c2/. The study has been approved by the scientific committee of Kurdistan Board for Medical Specialties (KBMS).

**Study design:** This is a prospective multicenter case control study. The cases were consecutive.

**Setting:** The study included two groups of participants, the first group was the case group and the second group was the control group. The cases were taken consecutively during 13 months (from March 2019 to April 2020) and for each case, one control counterpart was chosen in the same geographical and socio-demographical background. The patients were received and managed in different settings including academic, public and private hospitals.

**Inclusion criteria:** The inclusion criterion was a high school students with age between 16 and 20 years and studying in sitting position.

**Exclusion criteria:** The exclusion criteria were jobs other than being student, ages less than 16 or more than 19 years**,** other risk factors of PNS were excluded like prolong driving, being excessively hairy, obesity … etc. The information regarding one case was lost during data collection.

**Data collection and analysis:** Data were received from the questionnaire and the patient records. Microsoft Excel Version 2013 (Microsoft Corporation, Redmond, WA) was used for data collection, entry, and coding. Statistical Package for the Social Sciences (SPSS) Version 25 (IBM Corp, Armonk, NY) was used for conducting Data analysis. Continuous variables were compared by the Student's *t*-test, and discrete variables were analyzed using the χ2 test.

## 3 Results

This research was performed among 189 cases, (Control group: 95 cases, case group: 94 cases); 80 (42.3%) were male and 109 (57.7%) were female (Table 1). The age ranged from 16 to 19 years. About 8 (4.2%) participants were smokers; positive family history: 13 (6.9%) and those with diabetes mellitus were 3 (1.6%). There was no significant difference in both groups regarding basic features (Table 2). Among the control group 36 (37.9%) participants were used to study while sitting on a hard place whereas among the case group 62 (66%) cases were used to study while sitting on a hard place, the difference was statistically significant (<0.001) (Table 3).

**Table 1:** Basic characteristic of the participants.

Variable	N (%)
Sex	
Male	80 (42.3)
Female	109 (57.7)
Smoker	
Yes	8 (4.2)
No	181 (95.8)
DM	
Yes	3 (1.6)
No	186 (98.4)
FH of PNS	
Yes	13 (6.9)
No	176 (93.1)

**Table 2:** Basic characteristics of the two groups with comparison.

PNS N (%)			
Variable	No	Yes	P.Value
Sex			
Male	38 (40)	42 (44.7)	0.515
Female	57 (60)	52 (55.3)	
DM			
Yes	1 (1.1)	2 (2.1)	0.554
No	94 (98.9)	92 (97.9)	
Dermatological problem			
Yes	2 (2.1)	0 (0)	
No	93 (97.9)	94 (100)	0.157
Smoker			
Yes	3 (3.2)	5 (5.3)	0.461
No	92 (96.8)	89 (94.7)	
Excessive sweating			
Yes	11 (11.6)	10 (10.6)	0.837
No	84 (88.4)	84 (89.4)	
Being hairy			
Yes	17 (17.9)	18 (19.1)	0.824
No	78 (82.1)	76 (80.9)	
FH of PNS			
Yes	5 (5.3)	8 (8.5)	0.378
No	90 (94.7)	86 (91.5)

**Table 3:** Effect of various characteristics of the participants on development of PNS.

PNS N (%)			
Variable	No	Yes	P.Value
Hair color			
Black	56 (58.9)	60 (63.8)	0.752
Brown	31 (32.6)	26 (27.7)	
Blonde	8 (8.4)	8 (8.5)	
2 times or more baths per week			
Yes	83 (87.4)	77 (81.9)	
No	12 (12.6)	17 (18.1)	0.298
Study/day (Mean ± SD)	4.29 ± 1.68	4.00 ± 2.43	<0.001
Position			
Yes	88 (92.6)	91 (96.8)	0.200
No	7 (7.4)	3 (3.2)	
Friction			
Yes	40 (42.1)	30 (31.9)	0.147
No	55 (57.9)	64 (68.1)	
Hard place			
Yes	36 (37.9)	62 (66)	
No	59 (62.1)	32 (34)	<0.001
Medication			
Insulin	2 (2.1)	1 (1.1)	0.567
No	93 (97.9)	93 (98.9)	

## 4 Discussion

PNS is a relatively common perianal disorder found among young population with age range of 17–25 years [8]. Kaymakcioglu revealed the incidence rate of the disease of about 70% at 20–30 year of age [9]. Until mid of the previous century, many authors thought that PNS would be a congenital disease, Patey and Scarff accredited with the first mention of acquired hypothesis. They stated that the condition is due to hair suctioning from the adjacent skin. According to their theory, PNS is a long term condition results from penetration of stiff, short hairs into the subcutaneous region with resultant long term low-grade infection [10] Yildiz et al. recorded a positive family history in 52.4% of cases and first-degree relative with an ingrown hair history of about 35.7% [8].

Farmers were the most frequent reported jobs among the cases of sacrococcygeal PNS (15%) followed by those with irregular occupations (13.3%) and Salespeople (11.9%) [11]. Barber is the most prevalent job among those patients complaining from PNS of areas other than Sacrococcygeal region [12]. Classically being a driver is one of the risk factors for developing PNS due to prolonged sitting [13].

In the study by Harlak and associates, people with excessive hair who sit down for more than 6 h per 24 h and those who take path room less than three times per 7 days are at increased risk of developing PNS by 219 folds than those without these two risk factors [11]. This finding was completely rejected by the current study, the duration of sitting and the number of path per week had no effect on the development of PNS. This may be due the fact that the population of this study had no excessive body hair, a factor might be the causative agent in the other studies.

In the current study, common PNS risk factors such as family history, excessive hairiness, smoking, diabetes mellitus, hair color, sweating, dermatological diseases and personal hygiene have been equalized between the two groups to determine the specific risk factors related to the students. This result revealed that the duration of study is not significantly related to the development of the disease while the nature of the place (being soft or hard) that seated on is a significant risk factor (sitting on hard place is significantly higher in the case group (66%) in comparison to the control group (37.9%), P-Value less than 0.001), There are crucial limitations to this study; (1) the sample size is small, (2) although the participants were collected from several centers but they were from the same city and there was no population diversity.

In conclusion, sitting on the hard places could be regarded as a risk factor of developing PNS among secondary school students.

## Source of funding

None is found.

### Provenance and peer review

Not commissioned, externally peer reviewed.

## CRediT authorship contribution statement

**Faruq H. Faraj:** Project administration, Validation, Resources, Supervision. **Hiwa O. Baba:** Formal analysis, Funding acquisition, Investigation, Software, Visualization, Writing - original draft. **Abdulwahid M. Salih:** Formal analysis, Investigation, Methodology, Visualization, Writing - review & editing. **Fahmi H. kakamad:** Conceptualization, Data curation, Formal analysis, Investigation, Methodology, Resources, Software, Validation, Visualization, Writing - original draft.

## Declaration of competing interest

None to be declared.
